
JOURNAL INFORMATION
==============================
NLM Title Abbreviation: J Child Orthop
Journal ID: J Child Orthop
NLM Journal ID: 101313582

Journal of Children's Orthopaedics

ISSN: 1863-2521
EISSN: 1863-2548
Publisher: SAGE Publications

ARTICLE INFORMATION
==============================
PMCID: PMC4353815
PMID: 25711753
DOI: 10.1007/s11832-015-0638-z
Article ID: 638
Article version: 1
Subjects: Abstracts

EPOS 34th Annual Meeting

Electronic publication date: 2015 Feb 25
Print publication date: 2015 Apr
Volume: 9
Issue: Suppl 1
First page: 57
Last page: 65
Copyright: © The Author(s) 2015
License: This article is distributed under the terms of the Creative Commons Attribution License which permits any use, distribution, and reproduction in any medium, provided the original author(s) and the source are credited.
License URL: https://creativecommons.org/licenses/by/4.0/

issue-copyright-statement: © The Author(s) 2015

==============================

## PP1

### Efficacy and safety of percutaneous reduction and sacroiliac screw placement: a review of pediatric patients at a single institution

Brian Sager[1], Grant Hogue[2], Adam Starr[1]

[1]University of Texas, Dallas, United States
[2]Boston Children's Hospital, Boston, United States

**Trauma/lower extremity**

*Purpose:* Percutaneous techniques for fixation of posterior pelvic ring injuries are well described in the adult literature, but there is scarce data for this technique in the pediatric population. Our aim is to review cases at a single institution and report on complications, operative data, and quality of reduction with a pelvic reduction frame.
*Methods:* An IRB approved retrospective review was performed of 20 consecutive pediatric patients with posterior pelvic ring injuries and instability that had percutaneous intervention by a single surgeon from 2007 to 2011. Inclusion criteria were age $\leq$18 years, and unstable posterior pelvic ring injury that required sacroiliac (SI) fixation. Data parameters reviewed include age, skeletal maturity, operative data, quality of reduction, post-surgical complications, and classification of fracture type using the Young Burgess system, Orthopaedic Trauma Association (OTA) Classification, and the Shore modification of the Torode System.
*Results:* Eight male and four female patients were treated with percutaneous fixation of the SI joint with a mean age of 12.5 years (1–18 years). All patients exhibited an immature Risser sign (0–4) and four patients had open triradiate cartilage. The most common fracture pattern was a Lateral Compression Type fracture in five cases. There were also four Vertical Shear, two Anterior-Posterior Compression fracture types, and one mixed mechanism fracture types. All injuries were OTA 61-C and Shore-Torode type 4. Average initial displacement was 9.4 mm. All measured postoperative films showed $<$2 mm of displacement, except for a single patient with 3 mm of postoperative displacement. When patients who had concomitant open interventions in the same setting were excluded the average estimated blood loss was 9.4 ml. The mean anesthetic time was 137 min and average length of stay was 14.5 days. Four patients eventually had screws removed. A single patient, who also sustained a head injury, was found to have painless heterotopic ossification. Average follow up was 18.8 months.
*Conclusion:* Percutaneous fixation of the unstable SI joint is a viable treatment option in the pediatric population and allows for stable fixation with minimal blood loss and minor complications. Parents and patients should be counseled that in addition to risks already outlined in the adult literature these patients are more likely to require hardware removal.
*Significance:* To our knowledge, the technique of fluoroscopically guided SI screw placement with a pelvic reduction frame has not been reported in the pediatric literature. Particular importance should be noted of the four patients in our cohort with open triradiate cartilage.

## PP2

### 39 Years survival free from hip replacement after combined epiphysiodesis and imhauser inter-trochanteric osteotomy for slipped capital femoral epiphysis

Giovanni Trisolino[1], Giovanni Luigi Di Gennaro[1], Gherardo Pagliazzi[1], Stefano Stilli[1]

[1]Rizzoli Orthopaedic Institute, Bologna, Italy

**Hip/lower extremity**

*Purpose:* The main concern in the management of SCFE remains the treatment of cases with moderate to severe slippage. In such cases, it is generally accepted that the deformity must be corrected. The main dilemma is represented by the choice between an intra-articular or an extra-articular procedure: theoretically the intra-articular osteotomy allows for the best correction of the deformity but it is technically demanding and at higher risk of AVN and chondrolysis; conversely an extra-articular inter-trochanteric osteotomy (ITO) is less difficult and at lower risk of early complications but inevitably leads to femoro-acetabular impingement (FAI), resulting in early osteoarthritis (OA). We report our experience in treating moderate to severe SCFE by combined epiphyisiodesis and Imahauser ITO, and analyse the factors affecting the cumulative survivorship of the procedure.

*Methods:* This is a single centre retrospective continuous study evaluating the clinical and radiological evolution of patients presenting with moderate to severe SCFE treated by ISF combined with Imhauser ITO. From 1975 to 2000 a consecutive series of 44 patients (52 hips; 8 bilateral) underwent combined epiphysiodesis and Imhauser ITO. There were 26 males and 18 females with an average age of 12.9 ± 1.9 years (range 9–17). The severity of displacement was measured on radiographs using the Southwick angle. All cases showed a Southwick angle >40° (mean 69° ± 16°; range 40°–90°). The cumulative survivorship was performed according to Kaplan and Meier with the end point defined as conversion to total hip replacement (THR).
*Results:* Six patients (6 hips; 11 %) had a follow up less than 2 years. Among them no post-operative complications occured. For the remaining 38 patients (46 hips; 89 %) the mean follow up was 21 ± 11 years (range 2–39). Three early post-operative complications were reported (1 AVN; 2 Chondrolysis). The cumulative 39 years survivorship free from THR was of 66.7 % (95 % confidence interval: 39.4–83.8 %). Age at surgery (hazard ratio = 2.041 per year older; *p* value = .006) and the post-operative onset of AVN or Chondrolysis (hazard ratio = 10.481; *p* value = .018) significantly affected the long-term prognosis.
*Conclusion:* The combined epiphysiodesis and Imhauser ITO is a safe and effective surgical option in moderate to severe SCFE, preserving the natural hip for at least 39 years in the majority of patients. Care must be taken in avoiding AVN or Chondrolysis. The age at surgery negatively affects the prognosis.

## PP3

### Intraosseous injections of rhBMP-2 induces periosteal bone formation in a mouse model of osteogenesis imperfecta

Tegan Cheng[1], Aaron Schindeler[1], Craig Munns[1], David Little[1]

[1]Childrens Hospital at Westmead, Sydney, Australia

**Basic science**

*Purpose:* Significant interest surrounds agents with potential to improve bone strength in Osteogenesis Imperfecta (OI). Recombinant human bone morphogenetic protein-2 (rhBMP-2) is a potent anabolic stimulus for bone. Sucrose acetate isobutyrate (SAIB) is a viscous phase-transitioning carrier which we have used to deliver rhBMP-2 for bone formation. We hypothesize that intraosseous injections of rhBMP-2 via SAIB will increase bone on the periosteal surface, in turn increasing bone strength in a mouse model of OI.
*Methods:* Tibial reaming was performed on transgenic mice with a mutation in the *Col1a2* gene (OI) that exhibit a weak bone phenotype, as well as their wild type littermates (WT). Reamed tibias were then given intraosseous injections of either saline, or 5 µg rhBMP-2 in 20 µL of 80:20 SAIB:Ethanol. One group of each genotype received no treatment. Mice were culled 2 weeks post-injection. MicroCT was used to assess bone formation, and in two regions of interest (ROI) new bone on the periosteal surface was quantified.
*Results:* Reaming followed by intraosseous injections of either saline or SAIB admixed with rhBMP-2 significantly increased periosteal bone (new bone adjacent to the exterior cortex) detected in two ROIs in both WT and OI mice. In WT mice, rhBMP-2 induced bone volume (BV) increases of 91 and 179 % in the lower and upper ROIs respectively ($p < 0.01$ v Nil). For the OI animals, rhBMP-treatment resulted in increases in BV of 21 % ($p < 0.05$ v Nil) and 89 % ($p < 0.01$ v Nil) in the lower and upper ROIs, respectively. Sham treatment involving reaming and saline injection also produced an effect, though consistently less than rhBMP-2 treatment.
*Conclusion:* These data indicate that both Sham and rhBMP-2 treatments increased bone volume, however X-ray images and microCT showed more obvious periosteal bone formation with rhBMP-2. It is hypothesized that longer follow-up would allow for increased rhBMP-2 bone to form and that differences at a 4 week time point might be significant between rhBMP-2 and Sham. Increasing periosteal bone formation increases bone strength considerably as both torsion and bending strength are proportional to bone radius. Future studies will also examine interactions with bisphosphonates.
*Significance:* Intraosseous rhBMP-2 was able to increase significantly periosteal bone volume compared to nil treatment in both wild type and OI mice. While further study is required to validate this approach, the use of SAIB for rhBMP-2 delivery remains a promising approach for the treatment of the increased bone fragility associated with OI.

## PP4

### Factors leading to re-fracture in children with forearm shaft fractures treated by elastic intramedullary nails: analysis of 48 patients with hardware in situ

Marie Rousset[1], Antoine Samba[1], Mounira Mansour[2], Bruno Pereira[3], Federico Canavese[1]

[1]University Hospital Estaing, Clermont Ferrand, France
[2]University Hospital Estaing, Clemront Ferrand, France
[3]CHU Gabriel Montpied, DRCI, Clermont Ferrand, France

**Upper extremity only**

*Purpose:* Diaphyseal fractures of the forearm account for about 5 % of all children's fractures. Pediatric orthopaedic surgeons have to cope with potential complications. An occasional problem is re-fracture of the forearm with elastic intramedullary nail (ESIN) in situ. No published studies focusing on this problem exist. This study assesses the predisposing factors leading to re-fracture in patients with ESINs in place.
*Methods:* A retrospective analysis of consecutive patients with open physes, who had been treated with ESIN for forearm shaft fractures from 01/2011 to 12/2013 at our Institution, was conducted. This study assessed 48 consecutives children and adolescents with closed, displaced forearm fractures without neurovascular injury. Age, gender, weight and mechanism of injury were retrieved from charts. Type and location of fracture, nail size, radial and ulnar medullary canal diameter were assessed on digital antero-posterior and lateral radiographs of the forearm. Nail size/medullary canal diameter ratio (N/MC ratio) was calculated for all patients. Statistical analysis was made for dependency between re-fracture with ESIN in situ and N/MC ratio and confounding variables (age, weight, gender, mechanism of injury, type and location of fracture). Statistical significance was set at $p < 0.05$.
*Results:* Forty eight consecutive displaced forearm fractures (36 males, 12 females) were treated surgically. Re-fracture with intramedullary nail in place occurred in five patients (10.4 %). Occurrence of this complication was not significantly associated with N/MC ratio ($p > 0.05$) but was significantly associated with a young age ($p = 0.024$) in uni-variate and multi-variate analysis.
*Conclusion:* According to our findings, some precautions have to be taken when using ESIN in forearm shaft fractures, especially in patients younger than 7.5 years of age.
*Significance:* This work confirms that the younger patient is statistically at higher risk of re-fracture of the forearm when ESINs remain in situ.

## PP5

### The evolving state of acute pediatric septic arthritis and osteomyelitis

Derek Kelly[1], Don Franklin[1], Byron Stephens[1], Richard Smith[2], James Beaty[1], David Spence[1], Jeffrey Sawyer[1]

[1]Univ of TN-Campbell Clinic, Germantown, United States
[2]Univ of TN, Memphis, United States

**Musculoskeletal infections**

*Purpose:* To evaluate the evolving patterns of pediatric osteoarticular infection in concert with the emergence of community-associated MRSA as a prevalent pathogen.
*Methods:* The records of all patients treated at our institution for acute hematogenous septic arthritis and osteomyelitis between January 2005 and December 2011 were retrospectively reviewed for demographic, diagnostic, clinical, and radiographic data.
*Results:* Of the 240 patients who met inclusion criteria, 100 were diagnosed with MRSA osteoarticular infection, 51 were infected with MSSA, and 75 patients had no identifiable pathogen. The overall incidence of infection was 4.29 cases per 1,000 hospital admissions, with no significant annual variability. The mean age of MRSA patients was significantly lower (6.4 ± years), compared to MSSA patients [8.9 ± ($p = 0.002$)]. While MRSA patients experienced more febrile days and longer hospital stays than MSSA patients, there was no significant difference in admission laboratory values, surgical procedures, or long term complications. Subperiosteal abscess was evident in 51 and 45 % of MRSA and MSSA patients, respectively, compared to only 17 % of culture-negative patients ($p = 0.0001$). Surgical intervention was required in 87 % of MRSA patients and 84 % of MSSA patients. DVT was identified in 12 MRSA patients, 5 of whom subsequently developed septic PE. Eight MRSA patients developed chronic osteomyelitis, as opposed to only one case in MSSA patients. Intramuscular abscess was common to both groups, seen in 9/100 MRSA patients and 4/41 MSSA patients. Empiric antibiotic therapy consisted primarily of clindamycin and/or vancomycin, and was tailored according to microbial sensitivities.
*Conclusions:* Community-associated MRSA is a leading cause of pediatric osteoarticular infection, responsible for nearly half of all cases at our institution. Virulent strains of MSSA have now begun to produce similar clinical courses. Clinicians must maintain a heightened sense of awareness for the development of myriad morbid complications, particularly subperiosteal abscess requiring surgical intervention, and deep venous thrombosis with subsequent risk of septic pulmonary emboli.
*Significance:* An original publication from our institution in 2006 highlighted the increasing incidence and severity of community-associated MRSA bone and joint infections in children. While the incidence of these cases seems to have stabilized, patients infected with both MRSA and MSSA continue to experience serious clinical sequelae despite prompt recognition and appropriate antimicrobial therapy.

## PP6

### Could the rotational deformities of long bones be corrected by atemporary epiphysiodesis technique during growth? An experimental study

Mutlu Cobanoglu[1], Emre Cullu[1], Rahime Yaygingul[1], Figen Sevil Kilimci[1], Mehmet Kamil Ocal[1]

[1]Adnan Menderes University, Aydin, Turkey

**Basic science**

*Purpose:* The frontal plane deformities can be corrected by temporary hemiepiphsiodesis in growing children. But more invasive techniques like osteotomy and internal fixation are needed to correct the rotational deformities. The aim of this study is to evaluate the effect of temporary epiphysiodesis on rotational properties of the long bones during the growing period.
*Methods:* Forty-five New Zealand white rabbits (6 weeks old) were used. Two plates with four screws were placed in different directions (in oblique fashion relative to the physis and opposite to each other) on lateral and medial side of the proximal tibial epiphysis. There were three groups with 15 animals in each. Right tibias were used for control. Group 1 underwent surgery with plate and screw insertion. Group 2 underwent a sham operation with screw insertion only. Group 1 and 2 were observed for 4 weeks. Group 3 underwent surgery with plate insertion and the plates were removed 4 weeks after initial surgery to evaluate the rebound effect. Group 3 was observed four more weeks. All groups were euthanased at the end of the follow-up and bilateral hip disarticulation was performed and tibial rotation of both extremites was measured with computerized tomography. Rotation was defined as an angle between transcondylar axis (TC) and the distal tibial posterior axis (DTPA). The internal rotation of the TC according to DTPA was defined as negative value.
*Results:* The mean TC-DTPA was −20.28 ± 7.94 ($n = 13$) in right tibia and −2.91 ± 7.20 ($n = 7$) in left one in Group 1 ($p = 0.001$) and the mean TC-DTPA was −23.05 ± 4.91 ($n = 15$) in right and −25.80 ± 6.40 ($n = 15$) in left in Group 2 ($p = 0.167$), the mean TC-DTPA was −21.41 ± 6.44 ($n = 15$) in right and −9.50 ± 5.30 ($n = 14$) in left in Group 3($P = 0.000$). Although there was no difference in right TC-DTPA ($p = 0.523$) between all groups, there was statistically significant difference in left TC-DTPA ($p = 0.000$) between all groups. This result was due to the difference between Group 2 and both Group 1 and 3. Although the mean value of rotation in Group 3 was greater than Group 1, no significant difference was found ($p = 0.084$).
*Conclusion:* Derotation was obtained by temporary epiphysiodesis in tibia in this animal study. Although there was no statistically significant difference, rebound effect was seen after implant removal.

## PP7

### Safe drilling paths in the distal femoral epiphysis for pediatric medial patellofemoral ligament reconstruction

Cynthia Nguyen[1], Lutul Farrow[2], Raymond Liu[1], Allison Gilmore[1]

[1]Case Western Reserve Univ, Cleveland, United States
[2]Cleveland Clinic, Cleveland, United States

**Other/varia**

*Purpose:* Anatomic surgical reconstruction of the medial patellofemoral ligament (MPFL) is becoming increasingly popular for treatment of patellar instability in the skeletally immature population. The femoral attachment point of the MPFL is in close to proximity to the distal femoral physis. The safe zone angles for drilling the femoral epiphysis for graft placement have not yet been defined.
*Methods:* Twenty three cadaveric distal femoral epiphyses were scanned into high-resolution three-dimensional surface scan images. Using computer-aided design, the femoral insertion site of the MPFL was identified and marked. 8 mm diameter tunnels were drawn at varying angles to simulate the potential drill paths for placement of 6 mm interference screws with a 1 mm buffer on all sides. The distance from the MPFL footprint to where the drill paths violated the physis or the intercondylar notch was measured.
*Results:* Mean age was 11.2 ± 4.1 years. The percentage of samples where a 20 mm long drill tunnel at the given angles does not violate the physis or notch is shown in Table 1. For unsafe tunnels angled 5° distally or less, violation was almost always at the physis (129/131 tunnels, 98.4 %). For tunnels angled distal ≥15° but anterior 5° or less, there was substantial risk of violation of the notch (25/92 tunnels, 27.1 %).
*Conclusion:* Due to the undulating nature of the distal femoral physis, drilling into the epiphysis from the MPFL attachment site without angling risks damage to the physis. Angling the drill distal and anterior leads to less risk to the physis and notch respectively.
*Significance:* In order to minimize risk of trauma to the physis or notch, it is safest to angle the drill distal and anterior approximately 15°–20° in each plane from the MPFL attachment site.

Table 1 Number of safe 20 mm tunnels (%)

| Anterior inclination (°) | Distal inclination (°) | | | | |
|---|---|---|---|---|---|
| | 0° | 5° | 10° | 15° | 20° |
| 0° | 6 (26) | 13 (56) | 16 (61) | 16 (61) | 13 (56) |
| 5° | 5 (22) | 15 (65) | 17 (74) | 20 (87) | 18 (78) |
| 10° | 6 (26) | 14 (61) | 17 (74) | 21 (91) | 20 (87) |
| 15° | 6 (26) | 14 (61) | 17 (74) | 21 (91) | 22 (96) |
| 20° | 6 (26) | 14 (61) | 18 (78) | 21 (91) | 21 (91) |

## PP8

### Risk factors for developing dysplasia of the femoral trochlea in newborns

Christian Øye[1], Ketil Jarl Holen[1], Olav Andreas Foss[1]

[1]University Hosp. of Trondheim, Trondheim, Norway

**Basic science**

*Purpose:* A shallow femoral trochlea, as seen in trochlear dysplasia, is the single most important factor for patellofemoral instability. There is no consensus as to the etiology of trochlear dysplasia. The purpose of our study was to see if trochlear dysplasia is present at birth and to see if any specific risk factors for trochlear dysplasia could be detected.
*Methods:* By ultrasonography, both knees of 174 newborns were examined within 3 days after birth. In supine position, the most ventral part of the trochlea was exposed by flexing the knees above 45°. The Sulcus Angle (SA) of the femoral trochlea was defined. Demographic data for the newborns, like gender, weight, length, presentation, gestational age, hip status, congenital deformities and family history of patellar dislocations were registered. In the statistical analyses Mixed Linear Models were used to account for data dependency caused by knees and Generalized Mixed Models were used when analyzing dichotomous data with knees nested within children. The threshold value for dysplasia was defined by adding 2 SD to the mean SA value.
*Results:* The population was found representative with a gestational age of 39.1 weeks, mean birth weight of 3,476 g and mean birth length of 49.4 cm. The mean SA was 148°, the threshold value for dysplasia was ≥159°. 17 knees had a SA above this level. Among knees with dysplastic trochlea there was no association to parameters other than birth presentation. Breech presentation gave 16 times higher risk of dysplasia with an average 3.7° higher SA than a normal cephalic presentation. 12 out of 17 knees with dysplasia were in the breech position group, only 3 knees were found dysplastic after cephalic presentation. For the last two dysplastic knees the presentation was not known. As a last statistical step, the knee joint position was used as predictor of dysplasia. A strong correlation between an extended position of the knee and dysplasia of the femoral trochlea was detected with a 42 times higher risk of dysplasia for the extended knee. Children in the complete breech group did not have trochlear dysplasia.
*Conclusion:* Dysplasia of the femoral trochlea indicated by a high SA might be present at birth. Breech presentation seems to have a strong correlation to dysplasia and the position of the knees seems to be the crucial factor for developing trochlear dysplasia.

## PP9

### Long-term follow-up of arthroscopic treatment of discoid lateral meniscus in children

Jonathan Haskel[1], Tyler Uppstrom[1], David Dare[1], Scott Rodeo[1], Daniel Green[1]

[1]Hospital for Special Surgery, New York, United States

**Hip/lower extremity**

*Purpose:* The discoid meniscus, occurring almost exclusively on the lateral side, can lead to pain, popping, snapping, and decreased knee extension. The purpose of this study was to examine the long-term clinical outcomes of arthroscopic partial meniscectomy for the treatment of discoid lateral meniscus in children.
*Methods:* A previous study at our institution identified 27 consecutive patients who underwent arthroscopic meniscal saucerization by one of two surgeons between 1997 and 2002. These patients were included in this study if they were willing and able to complete the five outcomes questionnaires (IKDC Subjective Knee Evaluation, Kujala Scoring Questionnaire, Lysholm Knee Scoring Scale, Marx Activity Rating Scale and Tegner Activity Scale). Seven additional patients that were treated consecutively at least 10 years ago were also included in the study. Patients were also given the opportunity to receive a knee exam performed by one of the two treating surgeons. The exam was documented as per the IKDC knee examination protocol. Associations between outcome scores and discoid type, meniscal stability, location of instability, and age at time of surgery were identified.
*Results:* Of the 34 eligible patients (23 female, 11 male), 22 patients were contacted, and 21 agreed to participate. The average length of follow-up was 13.7 years, ranging from 10.3 to 16.6 years. Average age at the time of surgery was 9.3 years. Long-term follow-up revealed average IKDC, Kujala, and Lysholm scores of 82.87, 86.63 and 83.73, respectively. Additionally, average Marx and Tegner scores were 5.36 and 5.63, respectively. Stratifying the Lysholm scores revealed outcomes that were 45.4 % excellent, 18.2 % good, 27.3 % fair, and 9.1 % poor. The average IKDC Knee Examination score was A (normal). In total, 20.6 % (7 of 34) of eligible patients underwent a subsequent surgical procedure on the affected knee.
*Conclusions:* Numerous studies have demonstrated good to excellent short-term outcomes after arthroscopic treatment of discoid meniscus. At an average follow-up of nearly 14 years, our data suggests that clinical outcome scores decline over time. Compared to our previous study with 2-year follow-up, there is an increased rate of knee pain, mechanical symptoms, and functional limitations. Despite excellent post-operative IKDC examination scores, approximately 40 % of our patient cohort demonstrated relatively low Lysholm scores (fair and poor).
*Significance:* Long-term follow-up of young patients treated arthroscopically for symptomatic discoid meniscus demonstrate deteriorating patient reported outcome scores over time.

## PP10

### Inter- and intra-rater reliability of predictive models for the non-operative healing potential of stable juvenile osteochondritis dissecans (JOCD) knee lesions

Jonathan Haskel[1], Tyler Uppstrom[1], Elizabeth Gausden[1], Russell Meyer[1], Yong-Woon Shin[1], Joseph Nguyen[1], Daniel Green[1]

[1]Hospital for Special Surgery, New York, United States

**Hip/lower extremity**

*Purpose:* While juvenile osteochondritis dissecans (JOCD) lesions have greater healing potential than equivalent lesions in adults, only 50 % of JOCD lesions demonstrate radiographic healing after 6 months of non-operative treatment. Krause et al. (2013) and Wall et al. (2008) have described models to predict a patient's probability of healing with non-operative treatment based on patient and lesion characteristics. The Wall et al. nomogram incorporates normalized lesion length, normalized lesion width, and pain type, while the Krause et al. model includes normalized lesion width, patient age, and the size of concurrent cyst-like lesions. The purpose of this study was to determine inter- and intra-observer reliability of the predictive nomograms.
*Methods:* We retrospectively evaluated a consecutive series of children with open physes, who underwent non-operative treatment for stable JOCD lesions. Thirty-four patients (40 knees) were included in the study. At two time points at least 1 week apart, two medical students, two orthopaedic surgeons, and a radiologist made measurements of the JOCD lesions as described by Wall and Krause. These measurements, along with patient ages and pain type, were used to generate a point value for each lesion based on both the Krause and Wall models. Intra-class correlations (ICC) were calculated using the point value data obtained from the nomograms to determine inter- and intra-rater reliability.
*Results:* Based on the Landis and Koch (1977) threshold, we found near perfect intra-rater correlation for all raters in terms of individual OCD measurements, as well as total point score as calculated by both Krause (ICC = 0.843, 95 % CI 0.747–0.910) and Wall (ICC = 0.857, 95 % CI 0.773–0.917) methods. Additionally, amongst the five raters there was near perfect inter-rater reliabilities in total scores, as well as substantial to near perfect reliability in measuring the individual components of the scores (ICC = 0.780–0.929).
*Conclusions:* There is high inter- and intra-rater reliability for both the Krause and Wall point systems for predicting healing of JOCD lesions.
*Significance:* These models appear to be important tools for helping doctors and patients to predict reliably the healing potential of non-operative management of JOCD lesions.

## PP11

### The epidemiology of infections after pediatric spinal deformity surgery

Stephen Warner[1], Sean O'Brien[1], Andy Miller[1], Roger Widmann[1], Christine Salvatore[1], Stephanie Perlman[1]

[1]Hospital for Special Surgery, New York, United States

**Spine**

*Purpose:* Surgical site infection after pediatric spinal deformity surgery has significant adverse effects on the patient as well as substantial impact on the health care system. Whether the microbiology of these nosocomial infections has been altered by the advent of multidrug-resistant bacteria is unclear. The purpose of this study was to determine whether the epidemiology of surgical site infections after pediatric spinal deformity surgery at one institution has changed.
*Methods:* A retrospective review of pediatric patients who underwent spinal deformity surgery at a single institution from January 2000 to December 2012 was performed. Patients were included if they were under 21 years of age, underwent index surgery at our institution, and developed a deep surgical site infection during the study interval. Microbiologic findings were collected, in conjunction with data on patient demographics, underlying diagnosis, prophylactic and therapeutic antibiotics, surgical details, and postoperative outcome.
*Results:* During our study period, 951 patients underwent pediatric spinal deformity surgery at a single institution. Of these, 39 patients (4.1 %) developed a deep surgical site infection. Eight (21 %) of these patients were diagnosed with idiopathic scoliosis (IS) and 31 (79 %) were non-IS patients, including 22 (56 %) with neuromuscular scoliosis, 4 (10 %) with congenital scoliosis, and 5 (13 %) with syndromic scoliosis. Overall, the most common inciting pathogen was methicillin-sensitive *Staphylococcus aureus* (MSSA; 23 %), followed by *Proprionibacterium acnes* (*P. acnes;* 18 %), Coagulase-negative *Staphylococcus* species(18 %), and *Escherichia coli* (15 %). Polymicrobial infections were found predominantly in patients with neuromuscular scoliosis (13/16, 81 %, $p = 0.02$). In addition, 15 of the 17 (88 %) gram-negative infections occurred in neuromuscular patients ($p < 0.001$). Between 2000 and 2006 and between 2007 and 2012, MSSA was found in 4/18 (22 %) and 5/21 (24 %) of cases ($p = 1.0$), methicillin-resistant *Staphylococcus aureus* (MRSA)was found in (0/18) 0 % and 3/21 (14 %) ($p = 0.24$), and *P. acnes* was found in 3/18 (17 %) and 4/21 (19 %) ($p = 1.0$), respectively.
*Conclusions:* Despite changes in resistance patterns of bacterial infections in many areas, our data suggests the microbiology of surgical site infections in pediatric patients undergoing spinal deformity surgery has not significantly changed during the a 12 year period. In addition, these results confirm prior reports of significantly higher rates of gram-negative infections in patients with neuromuscular scoliosis and support further research to elucidate targeted perioperative antibiotics in this population.
*Significance:* Understanding the epidemiology of surgical site infections is critical for effective perioperative antibiotic prophylaxis as well as empiric antibiotic therapy.

## PP12

### A new method for assessing hip congruence after hip reduction in children with DDH

Paz Kedem[1], Saker Khamaisy[1], Ran Thein[1], David Scher[1], Roger Widmann[1]

[1]Hospital for Special Surgery, New York, United States

**DDH**

*Purpose:* The purpose of this study was to describe a quantitative method of assessing hip congruence, which uses MATLAB software to calculate the spherical centers of the acetabulum and femoral head from MRI.
*Methods:* Coronal PD sequences from post-surgical MRI studies of 24 patients (21 female, 3 male) who were treated for DDH were retrospectively reviewed. Study inclusion criteria consisted of patients between 0 and 18 months of age who were diagnosed with unilateral DDH, who underwent closed or open reduction of the hip and who received same-day post-operative pelvic MRI in a Spica cast. Using a least squares regression algorithm, the software calculated best-fit spheres for the acetabulum and femoral head of both the affected and unaffected hips. The program calculated the distance between these spherical centers and normalized this value to the diameter of the acetabulum to determine a Congruence Index (CI). The CI integrated three indices evaluated in the coronal, sagittal and axial planes. A CI = 1.0 indicated complete reduction and a CI = 0 indicated a complete dislocation. The inter-rater reliability of two raters was assessed in a random sample of 15 subjects. Post-operative radiographs and patient medical records were reviewed to identify patients who required a revision within 6 weeks following the procedure.
*Results:* The mean chronological age at the time of the procedure was 7.2 months (range 2–16 mo). Fourteen of the patients (58 %) required revision surgery within 6 weeks following primary procedures. The mean CI for hips that required revision (mean 0.51; range 0–0.8) was significantly smaller than the mean CI for hips that did not require revision (mean 0.79, range 0.61–0.93; p = 0.004). When evaluating the CI separately in all three planes, most of the subluxation was posterior. The average CI for unaffected hips was 0.94 (range 0.83–0.99). Inter-rater reliability was excellent, with weighted Kappa = 0.88 for dysplastic hips and weighted Kappa = 0.95 for unaffected hips. The calculated 95 % confidence interval included CI values ranging from 0.43 to 1.0.
*Conclusions:* We describe a novel, accurate and reliable method for calculating three-dimensional hip congruence, identifying residual subluxation, and indicating the direction of the subluxation. The calculated CI values reliably differentiated between hip reductions that required revision and those that did not require revision.
*Significance:* This novel, MRI-based method for assessing hip congruence may help predict the probability of success for both open and closed dysplastic hip reductions.

## PP13

### All-epiphyseal pediatric ACL reconstruction is superior to transphyseal ACL reconstruction in restoring axial stability and improved contact stresses during pivot-shift

Moira McCarthy[1], Peter Fabricant[1], Hamidreza Jahandar[1], Kyle Stone[1], James Boorman-Padgett[1], Frank Cordasco[1], Carl Imhauser[1], Daniel Green[1]

[1]Hospital for Special Surgery, New York, United States

**Hip/lower extremity**

*Purpose:* The complete transphyseal (CT) pediatric ACL reconstruction uses a vertical graft to minimize tunnel obliquity through physeal tissue, while the all-epiphyseal (AE) reconstruction avoids the physeal tissue completely by utilizing more horizontal sockets that theoretically result in more "anatomic" graft orientation. The purpose of this study was to compare directly CT and AE ACL reconstructions in order to determine if either technique is superior in restoring translational and/or rotational stability to the knee.
*Methods:* Ten fresh-frozen human cadaveric knees were tested with ACL-intact, ACL-deficient, and after each reconstruction method (CT and AE) using a robotic manipulator. Reconstruction order was randomized. Both reconstructions were fixed with identical suspensory fixation devices using an all-inside technique and 10 mm diameter hamstring grafts. Anterior stability was assessed with 134 N anterior force at 30° and 90° of flexion simulating the Lachman and anterior drawer exams, respectively. The pivot-shift exam was simulated by applying a combined 8 and 4 Nm of abduction and internal rotation, respectively, at 15° of flexion. Tibiofemoral motions were recorded and mean contact stresses generated within the tibiofemoral compartments were measured using contact stress transducers. Outcomes were compared across conditions of the ACL using ANOVA and Kruskall-Wallis tests for normally and non-normally distributed data, respectively.
*Results:* Both reconstructions had significantly decreased anterior translation, medial translation, and internal rotation compared to the ACL-deficient knee in response to a simulated pivot-shift. During the simulated Lachman and anterior drawer tests, both reconstructions restored anterior translation to that of the ACL-intact knee and offloaded the posterior aspect of the medial tibial plateau compared to the ACL-deficient knee. In response to the pivot-shift, however, the AE reconstruction decreased tibial internal rotation compared to the CT reconstruction and posteriolateral contact stresses compared to the ACL-deficient knee ($P < 0.05$), while contact stresses remained elevated following CT reconstruction.
*Conclusion:* While both AE and CT reconstructions reduced anterior translation and posteromedial contact stresses in response to an anterior load, the AE technique more effectively reduced axial rotation andposterolateral contact stresses during a simulated pivot-shift exam. These findings are likely due to the more oblique anatomic orientation of the AE graft, which makes it a more effective stabilizer to rotational and medial–lateral loads.
*Significance:* Surgeons should consider all-epiphyseal techniques with "anatomic" ACL footprints in skeletally immature patients.

## PP14

### Comparison of titanium cubic plate and transiliac bone graft for the treatment of planovalgus foot in children with cerebral palsy: a preliminary report

Muharrem Inan[1], Mustafa Incesu[2], Ilker Sarikaya[3], Ali Seker[4], Enis Yildirim[5]

[1]Istanbul University, Istanbul, Turkey
[2]Tepecik Training Hospital, Izmir, Turkey
[3]Cocuk Ortopedi Klinigi, Istanbul, Turkey
[4]Istanbul Medipol University, Istanbul, Turkey
[5]Sirnak State Hospital, Sirnak, Turkey

**Cerebral palsy**

*Purpose:* The planovalgus foot is a major source of disability in children with cerebral palsy. Surgical treatment can be chosen when conservative modalities are ineffective. Lateral column lengthening or subtalar arthrodesis are surgical options. We used a titanium cubic plate as a correction and fixation method in the calcaneal lengthening procedure to prevent reduction loss and recurrence. The purpose of this study is to describe this new fixation method and compare it to the conventional transiliac bone graft method.
*Methods:* Forty-five children (65 feet) with a diagnosis of CP underwent consecutive lateral column lengthening for symptomatic pes planovalgus foot with transiliac autograft (30 feet-group 1), titanium cubic plate (19 feet-group 2) or expandable titanium cubic plate (16 feet-group 3). The average age at surgery was 11.3 (range 5–29) years. There were 28 males and 17 females. Talus-first metatarsal angle (aTMA) and talonavicular coverage angle (TNCA) in anteroposterior radiographs, and talocalcaneal angle (L-TCA) in lateral radiographs were obtained pre and postoperatively.
*Results:* The preoperative mean aTMAs were 22.1° (±8.7°), 15.8° (±7.9°) and 17.2° (±8.5°) in group 1, 2 and 3, respectively. These values improved to 10.3° (±4.4°), 6.2° (±2.9°) and 7.3° (±3.3°) in group 1,2 and 3, respectively. The pre and postoperative mean TNCAs were 27.9° (±10.5°) and 7.9° (±3.6°) in group 1, 29° (±12.9°) and 8.5° (±3.3°) in group 2, 22.3° (±9.2°) and 8.2° (5.3°) in group 3, respectively. The mean pre and postoperative L-TCAs were measured as 41.6° (±6.6°) and 30.9° (±4.6°) in group 1; 40.3° (±6.8°) and 31.9° (6.6°) in group 2; 36.4° (±7.2°) and 27.9° (7.2°) in group 3. In all three techniques the differences between pre and postoperative values were significant. There was no significant difference between three methods.
*Conclusions:* Early results showed that this new technique has a comparable correction with calcaneal lengthening when compared with autograft. It may be the preferable technique for treatment of planovalgus foot in CP in patients with osteoporotic iliac bone and for whom allograft bone is not available.

## PP15

### Ponte osteotomies in the treatment of congenital scoliosis

Ted Sousa[1], Lindsay Andras[1], Elizabeth Joiner[1], Vernon Tolo[1], Paul Choi[1], David Skaggs[1]

[1]Childrens Hospital Los Angeles, Los Angeles, United States

#### Spine

*Purpose:* Congenital scoliosis is frequently treated with hemivertebrectomy or vertebral column resection to address rigid deformities. The purpose of this study is to determine if there are some congenital curves in which Ponte osteotomies can lead to sufficient correction.
*Methods:* Retrospective review of patients with congenital scoliosis who were treated between January 1996 to August 2014 with Ponte osteomies and posterior spinal fusion and a minimum 2 year follow up. Patients were excluded if they had prior instrumentation, isolated cervical deformity, or growing spine instrumentation.
*Results:* 76 patients were treated operatively for congenital scoliosis during this study period. Of these 38 underwent hemivertebrectomy and 5 underwent vertebral column resection. The remaining 26 (34 %) were treated with Ponte osteotomies and posterior spinal fusion without a vertebral resection. Of these 26 patients, nine were excluded for less than 2 year follow up. Of the 17 patients included in the analysis the mean preoperative Cobb was 66 (27–113). Mean operative time for patients treated with Ponte osteotomies was 286 min (range 148–448 min). Mean EBL was 500 mL (50–2,300 mL). An average of 12 levels (range 2–16 levels) were fused. A mean of 4 (range 1–7) Pontes were performed to achieve correction. Mean postoperative Cobb angle was 33° (range 3°–57°), representing 54 % (range 26–95 %) correction of the initial deformity. There was an average of 9° of correction obtained per Ponte osteotomy. Average follow-up was 3.8 year (range 2–9 years). Mean Cobb angle at final follow up was 34° (49 % average correction). One patient had a neurologic deficit, which resolved after a decompression and staged fusion. There were no other neurologic complications. During the follow up period three other patients had subsequent surgeries (1 for infection, 1 for proximal junctional kyphosis, 1 for prominent hardware).
*Conclusion:* We found that 34 % of surgically treated congenital spinal deformities in our institution could be treated with multiple ponte osteotomies with a favorable correction and safety profile.
*Significance:* This study demonstrates that Ponte osteotomies are an effective alternative to three column resections or hemivertebrectomy to obtain and maintain correction.

## PP16

### Decreasing pelvic incidence is associated with greater risk of cam deformity in femoroacetabular impingement

William Morris[1], Cody Fowers[1], Roger Yuh[1], Raymond Liu[1]

[1]Case Western Reserve Univ, Cleveland, United States

#### Hip/lower extremity

*Purpose:* Femoroacetabular impingement (FAI) is an increasingly recognized cause of hip pain in adolescents and young adults and preliminary studies have shown that it may be associated with increased risk of early osteoarthritis of the hip. Femoral and acetabular retroversion and slipped capital femoral epiphysis have been recognized as risk factors for formation of CAM deformity in FAI. A small, preliminary study from our institution demonstrated a possible association between CAM deformity and decreasing pelvic incidence. The purpose of this study was to validate the hypothesis that decreasing pelvic incidence is associated with increased risk CAM deformity.
*Methods:* 100 cadavers were selected from a historical collection with known pelvic incidences. Two matching cohorts were created: fifty subjects with high pelvic incidence (>60°) and 50 subjects with low pelvic incidence (<32°). Femoral anteversion and acetabular version were directly measured from each specimen bilaterally. Digital images were then obtained of the femora from an axial view perpendicular to the femoral neck to reproduce the view described by Notzli. Alpha angles were measured in all femora and CAM was defined as alpha angle >60°. Hip parameters were compared with t tests and incidence of CAM was compared with Chi squared test.
*Results:* CAM lesions were identified in 37/100 (37 %) femora in the cohort with pelvic incidence <32° and in only 21/100 (21 %) femora in the cohort with pelvic incidence >60° ($p = 0.013$). There was also a statistically significant difference in the mean alpha angles between the cohorts with pelvic incidence <32° (mean 49.7°, SD 10.6) and pelvic incidence >60° (mean 53.7, SD 10.7; $p = 0.008$). There was a trend towards pelves with decreased incidence to have relative acetabular retroversion (means 18.9 SD 7.2 versus 17.0 SD 6.2; $p = 0.05$). There was no statistically significant difference in femoral version between the two groups ($p = 0.559$).
*Conclusion:* Decreased pelvic incidence is associated with development of CAM deformity. We propose that subjects with decreased pelvic incidence would likely compensate during gait (to maintain lumbar lordosis) by tilting their pelvis forward, creating artificial anterior acetabular overcoverage. This would increase the risk for impingement during hip flexion and the formation of a reactive cam deformity.
*Significance:* An evolving understanding of the etiology of FAI may lead to changes in evaluation and management of adolescents and young adults with this pathology.

## PP17

### Searching for the ideal source for autologous chondrocyte implantation. New spotlight on GP chondrocytes

Ryszard Tomaszewski[1]

[1]GCZD, Katowice, Poland

#### Basic science

*Purpose:* Chondrocytes from the ipsilateral articular joint cartilage are the standard source of cellular material for ACI operations. Unfortunately, the harvesting procedure may have late effects on the joint causing morbidity, tenderness and sometimes stiffness. The aim of this project using New Zealand white rabbits was to study the use of physeal chondrocytes for restoration of articular cartilage defects during ACI procedure.
*Methods:* Fourteen New Zealand white rabbits, age 5 weeks, were selected. The physeal chondrocytes were harvested from the medial part of right tibias and than they were cultured. 22 days after the first procedure, the second operation was done during which a lesion in medial femoral condyle was made. The lesion was filled by autologous growth plate cultured cells. Two animals had no second operation because of death in one case and failed cellular culture in second. 60 days after the second surgery, the experimental animals underwent euthanasia. The samples from animals were collected and underwent morphological, macroscopic and microscopic evaluation and analyzed according to ICRS II score. Different types of histochemical and immunohistochemical stainings were used to visualize results of growth plate chondrocyte proliferation and matrix synthesis.
*Results*:

- Scaffold chondrocytes density was analyzed according to chondrocytes density in regenerated tissue and showed no relation between them.
- Macroscopic evaluation revealed change in color of regenerates.
- Aquired samples of the whole distal femur were prepared and evaluated after hematoxylin/eosin and Safranin O/fast green and staining according to the ICRS II histological scale showing good results of cartilagous healing in majority of cases.

*Discussion*:

- Physeal chondrocytes are useful as a good quality donor site for a chondrocyte culturing procedure forming hyaline like healing tissue after ACI reconstruction.
- There are paracrine and autocrine factors influencing chondrocytes density excluding scaffold chondrocyte density.
- GP chondrocytes give similar quality regeneration of articular cartilage defects to native cartilage chondrocytes.
- GP chondrocytes are more viable in culture having good potential for regeneration.

## PP18

### Neuromonitoring during excision of osteochondromas of the proximal fibula: is it worth it?

William Mackenzie[1], Mihir Thacker[1], Chris Marky[1], Kenneth Rogers[1], Anthony Sestokas[2], Anthony DiNardo[3]

[1]Nemours–AIDHC, Wilmington, United States
[2]Speciality Care, Inc., Lester, United States
[3]Speciality Care, Inc, Lester, United States

**Tumours**

*Purpose:* Osteochondromas of the proximal fibula are rare in children. Surgery may be needed in symptomatic patients who have pain and irritation of the peroneal nerve. The incidence of iatrogenic peroneal nerve injury during this procedure has been reported to be as high as 3 %. The purpose of this paper was to evaluate if intra-operative neuromonitoring of the peroneal nerve can improve patient safety and outcome.
*Methods:* We retrospectively reviewed surgical records and neuromonitoring data of consecutive patients who underwent resection of at least one osteochondroma of the proximal fibula at our institution between 2002 and 2014. Monitoring was provided by a single neuromonitoring group using a multimodality protocol that included spontaneous electromyography (EMG), transcranial motor evoked potentials (MEP) and somatosensory evoked potentials (SSEP). Outcomes measured were number of intraoperative alerts and new post operative deficits. Follow-up was to last visit as related to surgery.
*Results:* Thirty-one extremities (28 patients: males = 15, females = 13) with an average follow-up of 19 months were included. Three patients (9.7 %) had bilateral surgery. Pre-op neurologic symptoms were identified in 15 legs: (11 = numbness; 4 = tingling). Six (19 %) cases did not have neuromonitoring. Four (12.9 %) of the 25 monitored legs did not have normal baselines before surgery. The surgeon was alerted intraoperatively for 9/25 cases with changes in neuromonitoring. Alert types were EMG (3), EMG/MEP/SSEP (2), MEP (3), and SSEP (1). All legs returned to baseline after appropriate surgical intervention. In one case with abnormal baselines, excision of the osteochondroma did not result in improvement of the intraoperative signals and the peroneal nerve was decompressed with resultant improvement. At last follow-up, 12/15 patients had resolution of preoperative symptoms, 5 patients had altered sensation (three of these were pre-existent). No new motor deficits were identified postoperatively in all 25 cases monitored.
*Conclusion:* Our findings indicate a substantial number of cases (36 %) in which at least one neuromonitoring modality detected a potential problem intra-operatively and allowed timely intervention. Neuromonitoring may offer an effective way to address issues of avoiding injury to the peroneal nerve. The current protocol may be limited in its use for detection of sensory deficits.
*Significance:* Use of intra-operative neuromonitoring may help decrease the risk of damage to the peroneal nerve during excision of osteochondromas of the proximal fibula.

Open Access

This article is distributed under the terms of the Creative Commons Attribution License which permits any use, distribution, and reproduction in any medium, provided the original author(s) and the source are credited.

